# Colonic stem cell from severe ulcerative colitis maintains environment-independent immune activation by altering chromatin accessibility and global m^6^A loss

**DOI:** 10.1093/lifemedi/lnad034

**Published:** 2023-09-13

**Authors:** Chuandong Liu, Jie Li, Hua Jin, Qian Zhao, Fangle Li, Zurui Huang, Boyuan Mei, Wenxuan Gong, Xia Wang, Dali Han

**Affiliations:** Key Laboratory of RNA Science and Engineering, Key Laboratory of Genomic and Precision Medicine, Beijing Institute of Genomics, and China National Center for Bioinformation, Chinese Academy of Sciences, Beijing 100101, China; College of Future Technology, Sino-Danish College, University of Chinese Academy of Sciences, Beijing 100049, China; Key Laboratory of RNA Science and Engineering, Key Laboratory of Genomic and Precision Medicine, Beijing Institute of Genomics, and China National Center for Bioinformation, Chinese Academy of Sciences, Beijing 100101, China; School of Pharmaceutical Sciences, Tsinghua University, Beijing 100084, China; School of Pharmaceutical Sciences, Tsinghua University, Beijing 100084, China; Key Laboratory of RNA Science and Engineering, Key Laboratory of Genomic and Precision Medicine, Beijing Institute of Genomics, and China National Center for Bioinformation, Chinese Academy of Sciences, Beijing 100101, China; Key Laboratory of RNA Science and Engineering, Key Laboratory of Genomic and Precision Medicine, Beijing Institute of Genomics, and China National Center for Bioinformation, Chinese Academy of Sciences, Beijing 100101, China; School of Medicine, Tsinghua University, Beijing 100084, China; Key Laboratory of RNA Science and Engineering, Key Laboratory of Genomic and Precision Medicine, Beijing Institute of Genomics, and China National Center for Bioinformation, Chinese Academy of Sciences, Beijing 100101, China; School of Pharmaceutical Sciences, Tsinghua University, Beijing 100084, China; Key Laboratory of RNA Science and Engineering, Key Laboratory of Genomic and Precision Medicine, Beijing Institute of Genomics, and China National Center for Bioinformation, Chinese Academy of Sciences, Beijing 100101, China; College of Future Technology, Sino-Danish College, University of Chinese Academy of Sciences, Beijing 100049, China

**Keywords:** ulcerative colitis, colonic stem cell, chromatin accessibility, m^6^A; FTO

## Abstract

Ulcerative colitis (UC) is a chronic inflammatory disease of colon, which is characterized by cryptarchitectural distortion. Alternation of colonic stem cell (CoSC) contributed to the occurrence of UC, yet the regulatory mechanisms remain unclear. To investigate the dysregulation of transcriptional and post-transcriptional regulation, we performed RNA-seq, ATAC-seq, and m^6^A meRIP-seq analysis of the cultured CoSCs that were isolated from UC patients. The transcriptome analysis revealed distinct expression signatures of UC patients in mild and severe stages. We observed abnormal activation of immune and extracellular matrix-related genes in patients affected by severe UC. The chromatin accessibility at the promoter regions of these genes was also specifically increased in the severe stage. In addition, we identified that a global loss of RNA m^6^A modification in the severe stage was accompanied by higher expression of the m^6^A demethylase FTO. The aberrant activation of a large number of immune and extracellular matrix-related genes, including *IL4R*, *HLA-DPA1*, and *COL6A1*, was related to both the gain of chromatin accessibility and the loss of m^6^A in severe UC patients. Our finding revealed an environment-independent immune activation of CoSCs in UC and provided FTO as a potential therapeutic target.

## Introduction

Ulcerative colitis (UC) is one of the two major forms of inflammatory bowel disease (IBD), characterized by chronic inflammation and epithelial barrier disruption [1]. UC causes a persistent inflammation of the superficial colonic mucosa that may result in severe bleeding, ulcerations, toxic megacolon, and fulminant colitis [2, 3]. UC is a heterogeneous disease that can be classified by location affected (proctitis, left-sided colitis, proctosigmoiditis, and pancolitis), therapeutic effect (responder or non-responder), and severity (from mild to moderate to severe) [4, 5]. There is no definitive cure for UC, and the current treatment aims to reduce inflammation [4–6]. The general understanding of the disease is that immune dysregulation, genetic susceptibility, and environmental factors contribute to the progression of UC [7, 8]. However, the complex pathogenic mechanisms underlying UC are not fully understood.

The entire mucosal epithelium of the human intestine is renewed approximately every five days [9]. This process completely depends on the continuous self-renewal, proliferation, and differentiation of colon stem cells (CoSCs) [10]. CoSCs play a critical role in maintaining normal physiological structure, colon homeostasis, and regeneration after injury. In UC patients, CoSCs may be severely affected by a microenvironment characterized by chronic pathological inflammation, which contributes to colon physiological defects, abnormal regeneration, deterioration of the damage repair function, and even promotion of colitis-associated tumorigenesis [11]. It has been reported that CoSCs homeostasis is affected by external signal transduction and that it may be accompanied by changes in epigenetic factors [12, 13]. The exact pathological characteristics and regulatory mechanisms remain to be determined.

Recent studies have determined that DNA and histone epigenetic modifications together with chromatin accessibility regulate CoSCs self-renewal and colon integrity, thereby affecting normal intestinal homeostasis and regenerative capacity in UC [14–17]. *N*^6^-methyladenosine (m^6^A) mRNA modification is an important modulator for epi-transcriptional regulation, and can broadly affect mRNA splicing, translation, and degradation [18]. Emerging evidence reveals the essential role of m^6^A modification in many diseases’ progression and stem cell development [19]. Yet few studies have researched the global landscape of RNA m^6^A modification of CoSCs in UC patients.

Here, we isolated and cultured adult CoSCs *in vitro* from UC patients and healthy individuals following the protocol described by Wang *et al*. [20]. Using RNA-seq data from these cultured CoSCs, we observed the abnormal activation of immune and extracellular matrix-related genes, particularly dramatic in patients affected by severe UC. ATAC-seq reveals a strong state-specific promoter opening, which confirms the observation of abnormal transcriptional activation. In addition, we used m^6^A meRIP-seq and found a generalized loss of m^6^A modification in severe UC cases, confirming the abnormal transcriptional state once again. Fluctuations in the expression of multiple m^6^A regulatory proteins contribute to the overall m^6^A loss, with the up-regulation of the eraser FTO being key. Since CoSCs maintain such epigenetic and transcriptional signatures after several generations of culture out of the intestinal microenvironment, we suggest that the continued aberrant activation of these genes is independent of the environment. The discovery of this imprinting-like phenomenon provides novel insights to uncover pathological mechanisms of UC and discover potential therapeutic targets.

## Results

### Genes involved in immunity and ECM-related pathways are abnormally activated in CoSCs in patients affected by severe UC

**Figure 1.**
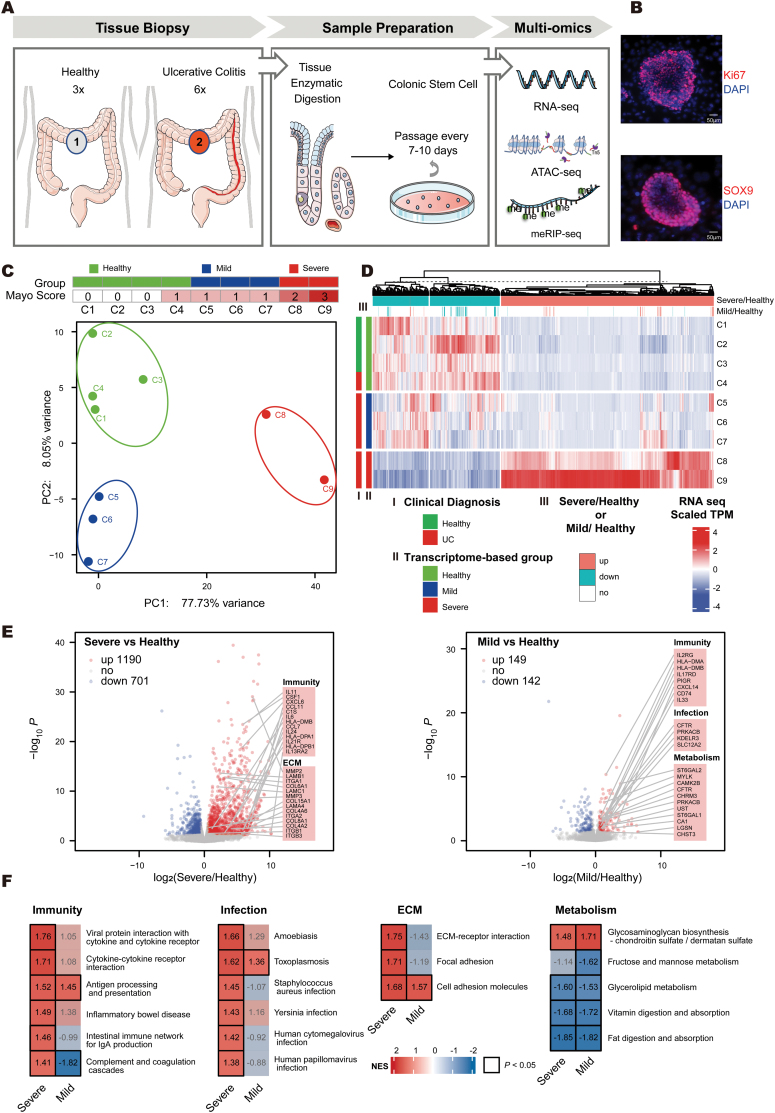
**Bulk RNA-seq revealed abnormal activation of immune and extracellular matrix-related genes in patients affected by UC disease, particularly evident in severe cases.** (A) Diagram showing the process used to acquire CoSCs and perform multi-omics sequencing. (B) Representative staining of CoSCs colonies stained with indicated antibodies for proliferative marker Ki-67 or colon epithelial stem cells marker SOX9 (red) merged with DAPI (blue). Scale bar, 50 μm. (C) PCA of top 500 variant genes in CoSCs from healthy individuals and UC patients. The upper panel shows groups determined by PCA and the corresponding Mayo scores. (D) Hierarchical clustering of dynamic genes distinguishing CoSCs from healthy individuals and UC patients. Heatmap colors represent *z*-score normalized transcripts per million (TPM). The bars represent group definition or transcriptional changes. (E) Left, volcano plot showing genes significantly altered in severe UC CoSCs compared to healthy individuals, with upregulated genes in red and downregulated genes in blue. Right, volcano plot of genes in mild UC CoSCs. Genes associated with the highlighted pathway are listed. (F) GSEA analysis showing differences in selected enriched pathways between mild and severe UC. Colors indicate the NES (Normalized Enrichment Score). Black squares around the values indicate *P*-value < 0.05.

To investigate whether colonic stem cells (CoSCs) are pathologically altered in UC conditions, we collected colonic biopsies from three healthy individuals and six UC patients (Table S1). Human CoSCs were isolated and cultured to maintain the commitment of a highly clonogenic, ground state form [20]. CoSCs obtained from healthy individuals and UC patients showed concordant expression of CoSCs marker SOX9 and proliferation marker Ki-67, while lacking typical differentiation markers such as intestinal goblet cell marker MUC2, as well as endocrine cell marker CHGA (Figs. 1A, 1B, S1A and S1B).

We performed RNA-seq for CoSCs derived from all individuals. To evaluate the differences and similarities between CoSCs from healthy individuals and UC patients, we performed principal component analysis (PCA) based on the expression matrix of the top 500 variant genes (Fig. 1C). Principal component 1 (PC1) and principal component 2 (PC2) were used to group these samples. Considering this UC patient-derived CoSC (C4) shows a limited difference of expression compared to three health samples, we treated this C4 sample as healthy CoSC and grouped this sample together with three healthy samples (mayo endoscopic score = 0) into a healthy group. We noticed that other UC patients could be further grouped into two clusters. Three patients with mild UC (Mayo endoscopic score = 1) were grouped together (mild group), and the remaining two patients with moderate or severe UC (Mayo endoscopic score = 2 or 3) were considered as the severe group. Hierarchical clustering of genes demonstrated that the transcriptional pattern of the severe group was distinct from the ones of the healthy and mild groups. In the severe group, we observed a large set of genes dramatically up-regulated compared to the healthy one, while fewer genes were up-regulated in the mild group (Fig. 1D). In summary, the diagnosis of UC based on transcriptomic data is more reflective of the essential differences between UC, especially severe UC, and healthy individuals at the molecular level than the traditional clinical mayo endoscopic score.

To explore the molecular states of CoSCs in healthy, mild, and severe groups, we performed differential gene expression analysis. Patients in the mild group showed 149 up-regulated genes and 142 down-regulated genes, while patients in the severe groups had 1,190 up-regulated genes and 701 down-regulated genes, revealing that the transcriptome of CoSCs from the severe group was dramatically altered (Fig. 1E). We inspected the functional enrichment of the DEGs in mild and severe CoSCs, and performed GSEA analysis of the KEGG pathways. We identified a number of significant functional terms (*P* < 0.05) in the severe group but not in the mild one, including inflammatory bowel disease, ECM–receptor interaction, complement and coagulation cascades, JAK-STAT signaling pathways, and Wnt signaling pathways (Figs. 1F and S1C), all of which support the severity of the clinical state.

We observed significant changes in antigen processing and presentation, as well as in multiple metabolic-related terms, including glycosaminoglycan biosynthesis and fat digestion and absorption, in both mild and severe CoSCs. Gene from several immune-related pathways, such as the TNF signaling pathway, TGF-β signaling pathway, and IL-17 signaling pathway, were more significantly up-regulated in the severe group than in the mild group, even in the presence of the TGF-β inhibitor SB431542 in the culture medium. Such results suggest that there is a general immune response in CoSCs and that the progression of the disease is gradual. We hypothesize that, because of the disruption of normal intestinal epithelial structures and the creation of cavities, cells normally protected by mucosa become susceptible to microbial and environmental factors, which may explain why immunity-related genes are abnormally activated and the ECM-related pathways are enriched in CoSCs. In addition, these analyses show that CoSCs obtained from both severe and mild UC patients show expression signatures of inflammatory states, which relate to the immune microenvironment, and maintain this signature even after five passages of *in vitro* cultures.

### CoSCs in mild and severe UC patients exhibit unique chromosomal accessibility signatures

**Figure 2.**
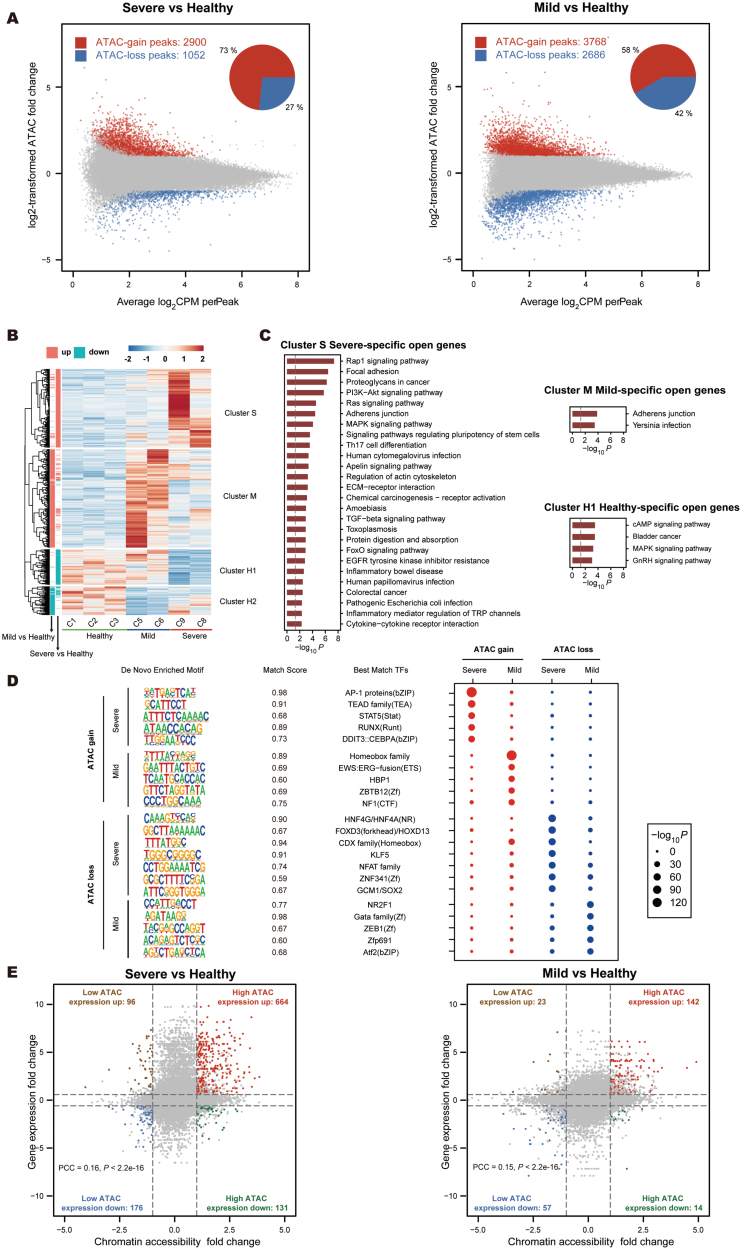
**Chromatin accessibility of immunity and ECM-related genes at promoter regions.** (A) Scatter plots comparing ATAC-seq signal at promoter regions in severe (left panel) or mild (right panel) UC CoSCs to that in healthy CoSCs. Pie charts show the proportion of genes with up- or down-regulated ATAC-seq signaling. Up-regulated genes are marked in red and down-regulated ones in blue. (B) Heatmap showing ATAC peaks specific to mild and severe UC patients identified by hierarchical clustering. Four ATAC-peaks groups are identified. (C) Enriched KEGG pathways of genes regulated by ATAC peaks from each cluster values are negative log_10_ transformed *P*-values. (D) Left, SeqLogo plot showing the top 10 enriched motifs found in ATAC-gain or ATAC-loss peaks in severe and mild CoSCs. Right, dot plot showing the statistical significance of all the top enriched motifs at ATAC-gain or ATAC-loss peaks in severe and mild CoSCs. The size of the dots refers to the negative log-transformed *P*-value. (E) Scatter plots showing the relationship between fold changes of ATAC signal at promoter regions and fold change of expression level in severe (left panel) or mild (right panel) UC CoSCs compared to healthy individuals.

To further understand the abnormal activation of immunity and ECM-related pathways in UC CoSCs, we generated chromatin accessibility maps with ATAC-seq to evaluate whether chromatin landscape changes in UC CoSCs contributed to the abnormal activation. We used differential accessibility analysis to identify regions that had significantly gained or lost accessibility in severe and mild UC CoSCs compared to healthy CoSCs. The results revealed large-scale changes in chromatin accessibility in both disease stages (Fig. 2A). The ATAC-gain or ATAC-loss regions can respectively indicate activation or inactivation tendency in transcription of genes present in those regions. Among them, the up-regulation of transcription due to ATAC-gain was more dramatic in severe CoSCs compared to mild ones (Fig. S2). It is worth noting that 73% of these differential regions gained accessibility in the severe UC group (Fig. 2A), consistent with our observations about gene expression in severe UC that there are more up-regulated (1190) genes than down-regulated genes (701) (Fig. 1D). Taken together, these analyses reveal large-scale genome activation and increased chromatin accessibility in severe UC CoSCs.

By hierarchical clustering of dynamic ATAC peaks, we identified clusters of open chromatin regions that are specific to severe UC (cluster S), mild UC (cluster M), and healthy CoSCs (cluster H1 and H2; Fig. 2B). Severe UC CoSCs shared a limited number of ATAC-gain peaks with mild ones (572/2,900). In addition, we performed KEGG enrichment analysis for genes associated with these clusters. Consistently with our previous observations, immunity and ECM-related pathways are specifically enriched in cluster S, but not in cluster M (Fig. 2C), which indicates the over-activation of genes in severe UC may be specially regulated by the chromatin accessibility of promoter regions. These results suggest that different regulators may mediate transcription changes in different disease stages. ATAC-gain peaks of severe UC were enriched in binding motifs of JunB, AP-1, and TEAD4 (Fig. 2D), which were reported to be related to inflammatory diseases [21, 22], while ATAC-gain peaks of mild UC were enriched in motifs of the homeobox family, which were reported to be related to development [23, 24]. In addition, ATAC-lost peaks of both severe and mild UC were enriched in motifs of stemness-related transcription factors, including KLF family, SOX family, and GATA family (Fig. 2D).

In conclusion, these results suggest that chromatin accessibility of immune and ECM-related genes is specifically turned on in the late stages of UC, leading to continued abnormal activation of these genes. Yet the positive correlation coefficient between changes in chromosome accessibility and changes in gene expression was between 0.1 and 0.2 in UC CoSCs (Fig. 2E), which suggests there may be other key factors regulating transcription.

### A global m^6^A loss occurs in severe CoSCs partially accompanied by higher expression of FTO

ATAC-seq demonstrated chromatin accessibility at promoter regions has stage-specific characteristics and other regulatory factors may be involved in the aberrant activation of immune and ECM-related genes in UC patients. RNA m^6^A modification is the most common and abundant mRNA modification and is involved in the mRNA biological role determination. To dissect the role of RNA m^6^A in UC, we mapped the m^6^A methylomes with m^6^A meRIP-seq in all three healthy individuals, two mild UC patients, and two severe UC patients.

**Figure 3.**
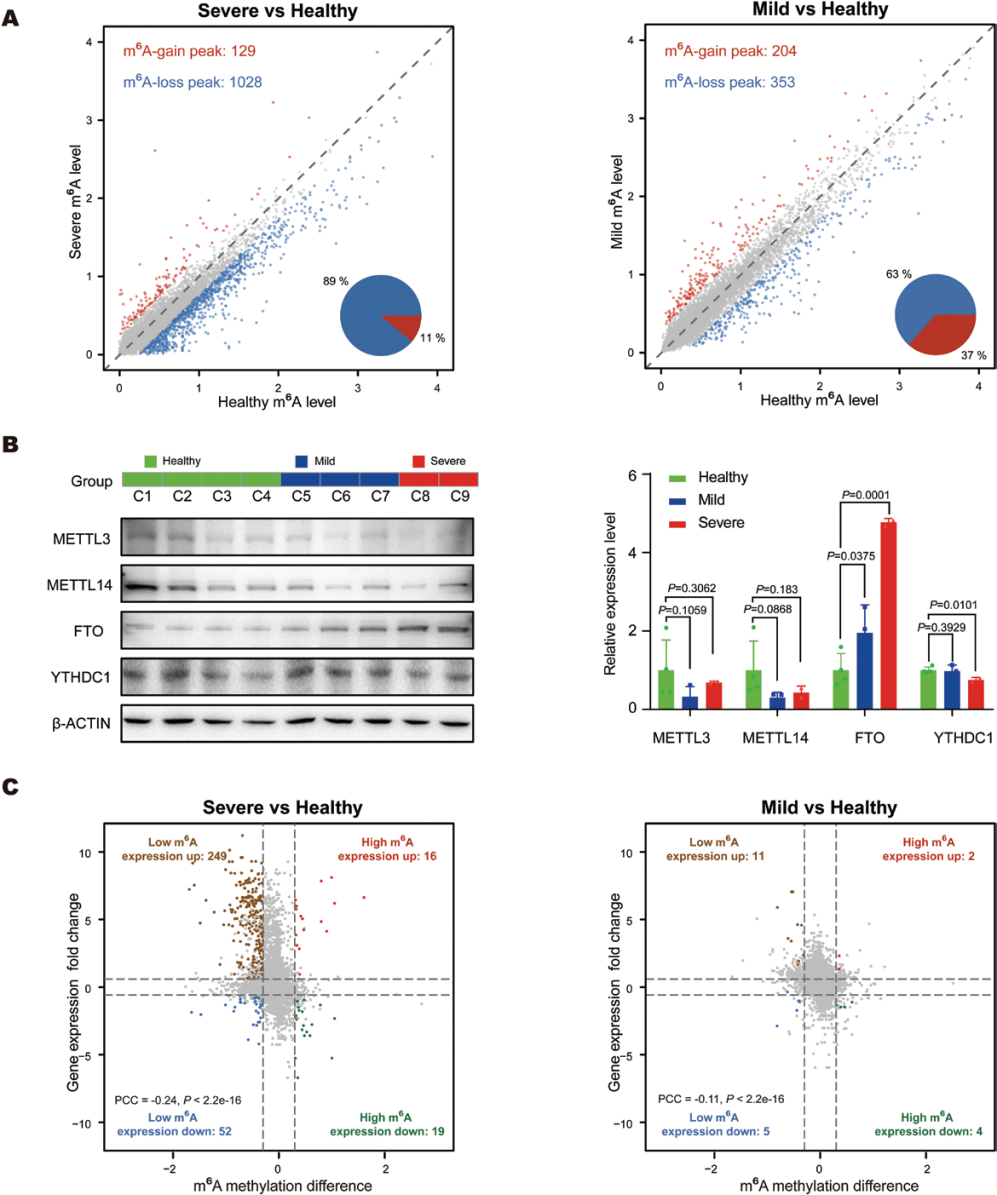
**RNA m**^**6**^**A levels showing a global down-regulation in severe UC CoSCs accompanied by higher expression of FTO.** (A) Scatter plots comparing m^6^A in severe (left panel) or mild (right panel) UC CoSCs to that in healthy CoSCs. Pie charts show the proportion of the genes with up- or down-regulated m^6^A signaling. Up-regulated genes are marked in red and down-regulated ones in blue. (B) Left, immunoblotting of METTL3, METTL14, FTO, and YTHDC1 in healthy individuals, mild and severe UC patients. Right, bar plot representing the differential expression levels of indicated protein calculated from the grayscale values of the protein bands shown in the left panel. Error bars indicate the standard deviation. *P-value < 0.05; **P-value < 0.01. (C) Scatter plots showing the relationship between m^6^A signal difference at gene body regions and fold change of expression level in severe (left panel) or mild (right panel) UC CoSCs compared to healthy individuals.

As expected, the m^6^A peaks of all three groups exhibited enrichment of the canonical m^6^A motif GGACU, and were predominantly localized near the stop codon (Fig. S3A). We subsequently calculated the m^6^A ratio for each peak and identified differential methylated m^6^A peaks. By comparing severe CoSCs to healthy CoSCs, we identified 1,028 m^6^A-loss peaks, representing 89% of the differentially methylated m^6^A peaks, and found only 129 m^6^A-gain peaks (11%) in severe CoSCs (Fig. 3A). This global loss of m^6^A methylation was not observed by comparing mild CoSCs to healthy CoSCs, which identified a comparable number of m^6^A-loss peaks and m^6^A-gain peaks (353 vs 204). Quantification of the m^6^A level in served and healthy CoSCs also showed that m^6^A methylation on mRNA was globally reduced in CoSCs from serving UC patients (Fig. S3B).

We next investigated the reason why the level of RNA m^6^A generally decreases in severe UC. We examined the changes in the transcription level of m^6^A reader, writer, and eraser proteins with RNA-seq (Fig. S3C). We then selected methylase transferases METTL3 and METTL14, demethylase FTO and an m^6^A reader YTHDC1, which varied more significantly in UC CoSCs and the healthy ones, to perform Western blot. The substantial loss of m^6^A in severe disease indicates the fluctuations of m^6^A regulatory proteins. The results of the Western blot showed that the protein level of FTO increased with the aggravation of inflammation, while METTL3 and METTL14 had an opposite trend (Fig. 3B).

Because m^6^A levels are closely associated with the decay and abundance of the m^6^A-marked transcripts, we next focused on the relationship between m^6^A levels and the expression level of the m^6^A direct-regulated transcripts in CoSCs. Compared to mild CoSCs, m^6^A loss in severe CoSCs leads to more dramatic expression up-regulation, which is consistent with global m^6^A loss in severe CoSCs (Figs. 3C and S3D). The changes in m^6^A and in expression levels are negatively correlated (Fig. 3C). Yet post-transcriptional regulation is insufficient to fully explain the expression changes we observed, suggesting that more regulatory factors and regulatory mechanisms need to be investigated. These results collectively confirm that in severe CoSCs m^6^A regulatory proteins generally increase the level of eraser protein and reduce one of the writer proteins through a coordinated transcriptional regulatory mechanism. This mechanism is responsible for the global m^6^A loss in severe UC.

### Synergistic effects of chromatin accessibility and m^6^A modification lead to aberrant activation of immune and ECM-related genes

**Figure 4.**
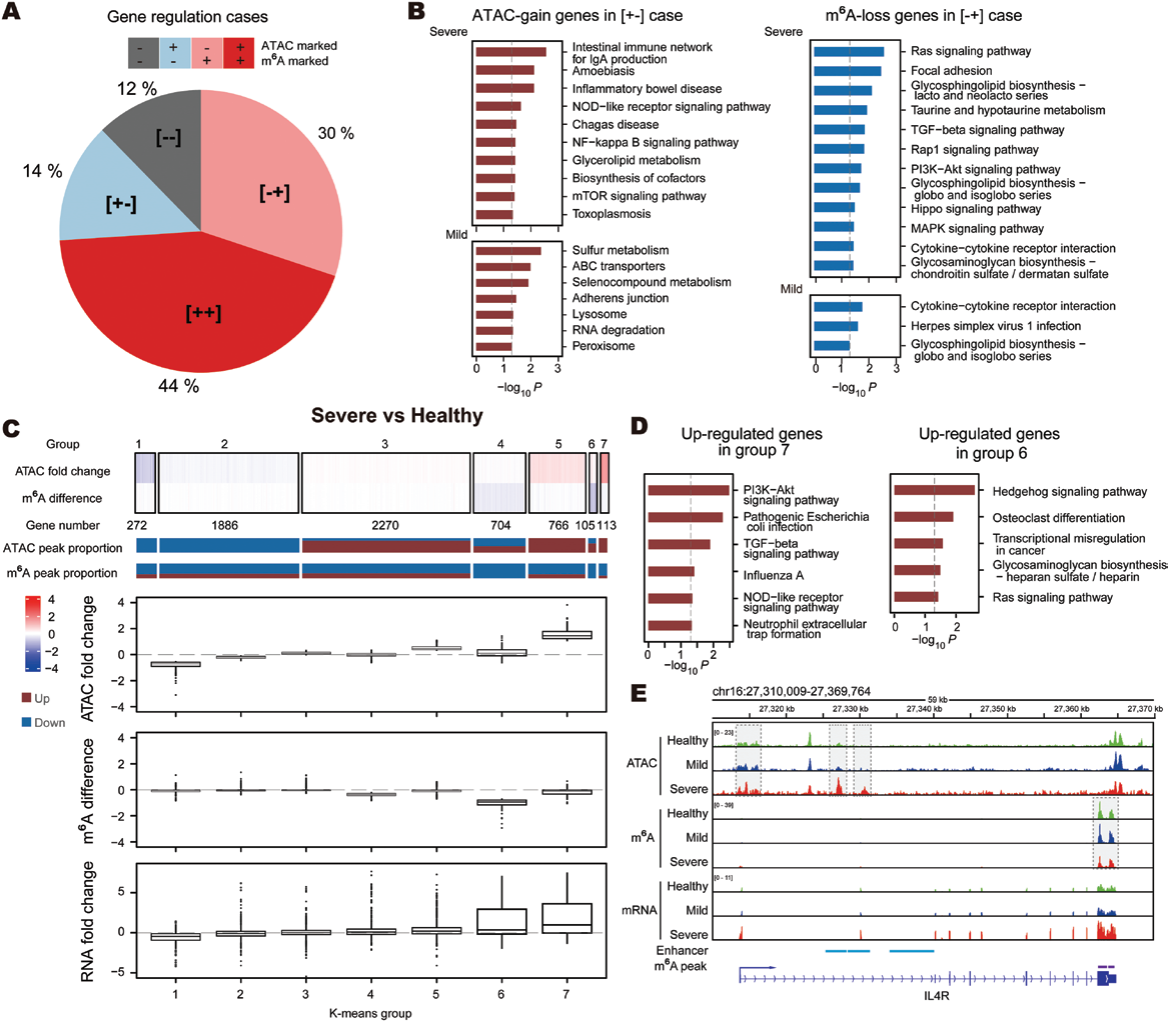
**RNA m**^**6**^**A and chromatin accessibility determine the abnormality of the downstream transcriptome in UC patients.** (A) Pie chart showing the ratio of the four types of genes. Promoter open +: promoters with ATAC signal; m^6^A marked +: genes with m^6^A signal at gene body region. (B) Bar plots showing the pathways enriched in severe and mild CoSCs compared to the healthy ones. Left panel refers to the ATAC-gain genes in [+−] cases. Right panel refers to the m^6^A-loss genes in [−+] cases. Values are negative log_10_ transformed *P*-values. (C) Genes co-regulated by m^6^A and chromatin accessibility at promoter regions classified into seven groups by k-means clustering shown in the upper heatmap. The middle bar graph shows the percentage of up- or down-regulation of m^6^A or ATAC signals on genes in each clustering group. The boxplot below presents the difference values of m^6^A signal and fold changes of ATAC signal and RNA expression in each group of genes in severe UC patients compared to the healthy ones. (D) Bar plot showing the pathways enriched by the genes of the selected groups in severe and mild UC patients compared to healthy individuals on genes with open promoters and m^6^A signal. Values are negative log_10_ transformed *P*-values. (E) IGV tracks displaying RNA and m^6^A abundances of IL4R transcripts in CoSCs.

Because neither chromatin accessibility nor m^6^A modification can fully explain the changes in gene expression, we counted all genes and then grouped them into four regulatory types: 44% having both ATAC peaks in the promoter regions and m^6^A peaks in the gene body region ([++] cases), 30% regulated by m^6^A only ([−+] cases), 14% regulated by the promoter openness only ([+−] cases), and 12% not regulated by either of these epigenetic factors ([−−] cases; Fig. 4A).

In the ATAC-peak only genes ([+−] cases), the ATAC-gain genes were more significantly enriched in immune-related pathways, such as the intestinal immune network for IgA production and inflammatory bowel disease, in severe CoSCs compared to mild ones. Between m^6^A-peak only genes ([−+] cases), in severe CoSCs the m^6^A-loss genes were enriched in immune and ECM-related pathways, such as TGF-beta signaling pathway and focal adhesion, while in mild CoSCs they were m^6^A enriched to some extent in immune-related pathways such as cytokine−cytokine receptor interaction and herpes simplex virus 1 infection (Figs. 4B and S4A), suggesting there is a progressive increase in inflammatory stress of stem cells as UC disease progresses. In severe CoSCs, the pathways enriched in ATAC-gain and m^6^A-loss regulated genes in severe CoSCs were similar to those enriched in genes up-regulated, consistently with the fact that ATAC-gain and m^6^A-loss tend to up-regulate the expression of target genes. The differences in ATAC-only and m^6^A-only regulated genes may be due to chromatin accessibility and associated transcription factors being more stage-specific, while the effect of m^6^A modification on RNA decay is more general.

We focused on genes with chromosomal accessibility and m^6^A dual regulation ([++] cases). After sorting gene expression by fold change, we found that, especially in severe CoSCs, genes with simultaneous ATAC-gain and m^6^A-loss signals underwent a more significant activation (Fig. S4B). Then we performed K-means clustering based on the difference of ATAC and m^6^A between UC CoSCs (severe or mild) and health CoSCs of these genes with dual regulation and classified them into seven groups (Figs. 4C and S4D). We found that severe CoSCs had more dramatic fluctuations of chromatin accessibility and m^6^A modification, resulting in more pronounced downstream transcriptional changes. Gene groups 6 and 7 in severe CoSCs, characterized by the leading dominant promoter opening and m^6^A loss, and thus the most dramatic up-regulation of transcription level, were of particular interest. For these two groups, ATAC-gain and m^6^A-loss generally occurred together, but not in equal measure, suggesting that the regulation of the target genes by chromatin accessibility and m^6^A is not a simple coordinated effect. Immune- and ECM-related pathways were also enriched in these groups (Fig. 4D). For example, IL4R from group 7 and ADAMTS14 from group 6 in severe CoSCs displayed both gained chromatin accessibility at promoter and enhancer regions and decreased m^6^A abundance at their 3ʹ UTRs and had significantly increased RNA levels in severe UC (Figs. 4E and S4D). IL4R has been widely studied in association with colon tumors [25–27], which gave us vital help in understanding why severe UC might develop into colon tumors [28]. Taken together, the synergistic effect of promoter region opening and m^6^A loss in severe CoSCs led to high-level transcriptional activation.

## Discussion

**Figure 5.**
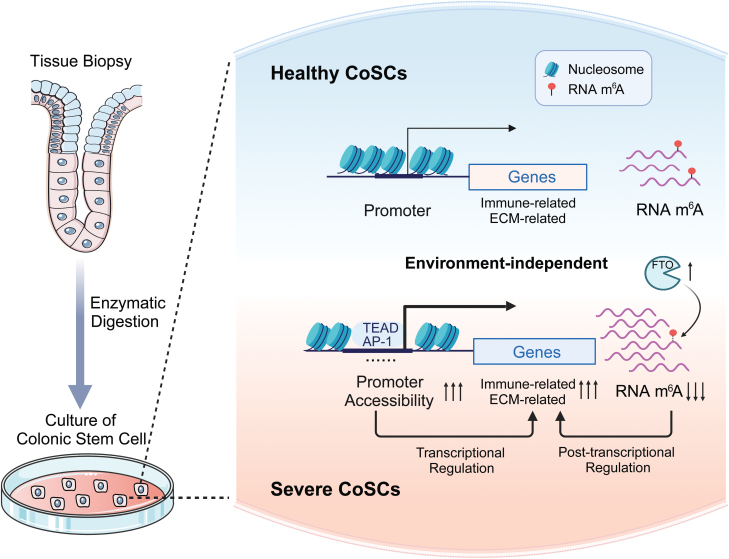
**Graphical summary.** The synergistic effect of chromatin accessibility and RNA m^6^A modification leads to the aberrant activation of immune and ECM-related genes in serve CoSCs.

Comparing the transcriptomes of severe and mild UC patients and healthy individuals, we identified a large-scale abnormal up-regulation of immune- and ECM-related genes in the colon stem cells of severe patients. This suggests that the absence of barriers and the strong inflammatory response micro-environment induced by ulcers in the colon of UC patients [1, 29] affects colon stem cells. On one hand, contact with colonic microbes activates the activation of immune-related pathways in colonic stem cells to defend against pathogenic attack, while the up-regulation of MHCII genes (e.g., HLA-DMB, HLA-DPA1, and HLA-DPB1) can help immune cells such as T cells to regulate intestinal stem cells to promote colonic stem cell renewal and differentiation [30]. On the other hand, the up-regulation of ECM-related genes may compensate to some extent for the mucosal barrier deficiency to reduce the damage suffered by colonic stem cells. These aberrant up-regulated genes potentially give colon stem cells a competitive advantage in a strong inflammatory response environment from severe UC patients. ATAC-seq data demonstrated that the immune response-related transcription factor AP1-JunB is stage-specifically bound to the promoter region of immune-related genes in severe patients. In contrast, at the level of post-transcriptional regulation, m^6^A shows a global loss in severe patients with a consequent reduction in RNA degradation rate. We also identified 272 genes with both elevated chromatin accessibility and reduced levels of RNA m^6^A, of which both epigenetic changes contributed to the high level of gene expression in the severe condition (Fig. 5).

Recent studies have identified the specific crosstalk between RNA modifications and chromatin accessibility and revealed that m^6^A machinery could exert pleiotropic effects on chromatin state directly or indirectly [31, 32]. Mechanistically, m^6^A machinery may facilitate shaping genomic architecture and maintaining genomic stability by regulating gene transcription [33], DNA damage repair [34], or histone modification [35] processes. Here, to explain the global m^6^A loss in severe disease cases, we detected levels of main m^6^A modification regulators. Our data indicated significant up-regulation of m^6^A demethylases FTO in UC patients, especially in severe cases, as well as a slight down-regulation of m^6^A methylases METTL3 and METTL14. FTO has exhibited a significant association with chromatin regulations: On the one hand, FTO can directly regulate histone modifications and chromatin states in the nucleus [33, 36]; On the other hand, FTO levels affect RNA modification of several transcription factors transcripts (e.g., STAT3 [37], JunB and C/EBPb [38], FOXJ1[39], FOXO1[40] and thus affect the stability and chromatin accessibility of their binding sites. Based on the multi-omics analysis, we observed that CoSCs in mild and severe UC patients exhibit unique transcription factor patterns and chromosomal accessibility signatures, which are partially cooperatively organized by their dysfunction of m^6^A modification. Such a phenomenon can help to explain how the CoSCs are modulated by RNA m^6^A modification machinery to maintain a homeostasis state during chronic inflammation.

Although recent studies reported that m^6^A regulatory proteins METTL3 [41], METTL14 [42], and YTHDF1 [43] affect intestinal epithelial cell homeostasis, our work delineated the disordered expression and dysfunction of FTO in CoSCs under physiological inflammation conditions. Since FTO tends to be highly expressed in a variety of inflammatory conditions and cancers and is considered a potential therapeutic target. Certain transcripts often accumulate to disturb the cellular system by way of FTO-dependent RNA processing and metabolism. Therefore, the development of FTO inhibitors could be exploited as a promising strategy for clinical application to elicit compensatory mechanisms through autoregulatory feedback loops of m^6^A modification machinery. Promising progress in developing small-molecule FTO-selective inhibitors is continuing to be made, such as Entacapone [40], FB23 and FB23-2 [44], CS1/2 [45], Dac51 [38]. Our recent work that identified the role of FTO in promoting tumor glycolysis and restricting T-cell responses, and developed the novel FTO inhibitor Dac51 which can increase CD8^+^ T-cell infiltration in tumors as part of the host adaptive immune response [38]. In this work, CoSCs profile (showing specific immune- and ECM-related gene expression) from UC patients is similar to what was observed in immune and epithelial cells identified by the ligand-receptor analysis, suggesting that the FTO dysregulation may lead to a disordered interaction between CoSCs and their microenvironment in UC condition. FTO inhibitors may represent a potential function to alleviate the pathological changes of chronic inflammation imprints and immune disorders in CoSCs, maintaining their pluripotency and differentiation balance under inflammatory bowel disease.

Collectively, our work demonstrates the critical role of chromatin accessibility and RNA m^6^A modification in maintaining the aberrant activation of CoSCs in severe UC patients. In particular, eliminating constraints by treating UC CoSCs with FTO inhibitors will be informative to fully understand the molecular mechanism and highlight their therapeutic potential for future drug development.

## Research limitations

One limitation of our study is the small sample size, especially for severe UC cases. Nevertheless, our findings are compatible with the conclusions of existing IBD literature [41, 46], which gives us reasons to believe that our observations are not influenced by outliers. Future studies should increase the sample size at different UC stages to further validate our results.

In terms of gene expression regulation, ATAC-seq and m^6^A meRIP-seq data alone are not sufficient to fully explain the observed transcriptional aberrant activation. For example, previous work has identified a reduction in HNFα in UC patients [47] and the therapeutic potential of AP-1 inhibitors [48], indicating that the ChIP-seq of these transcriptional factors might figure out other vital synergetic factors. We also recommend more studies be carried out on other transcription factors, histones, RNA binding proteins, and chromosomal 3D structures to better understand the mechanisms involved in transcriptional regulation.

## Materials and methods

### Isolation and culture of human colon stem cell *in vitro*

Colon biopsies from Non-IBD or UC patients were obtained under endoscopy at the General Hospital of the People’s Liberation Army, the Beijing Tsinghua Changgung Hospital, and the Peking Union Medical College Hospital. Sample collection and cell culture were performed following an existing protocol, with slight modifications [20]. The biopsies were placed in cold F12 media (Gibco) with 5% fetal bovine serum (HyClone) and subsequently minced into 0.2–0.5 mm^3^ sizes to a viscous and homogeneous appearance with a sterile scalpel. The minced tissues were digested with 2 mg·mL^–1^ collagenase type IV (Gibco) at 37°C for 30–60 min with agitation. Dissociated cells in suspension were then passed through 70-µm Nylon strainer (Falcon), centrifuged at 300 × *g* for 5 min at 4°C, and then washed four times in cold F12 media. Cells were seeded onto a feeder layer of lethally irradiated 3T3-J2 cells in SCM-6F8 media (Advanced DMEM/F12 supplemented with 100 ng·mL^–1^ human Noggin (Peprotech), 1 mM Jagged-1 (AnaSpec Inc.), 125 ng·mL^–1^ R-spondin1 (R&D systems), 2.5 mM Rock-inhibitor (Calbiochem), 2 mM SB431542 (Cayman chemical), 10 mM nicotinamide (Sigma-Aldrich), 100 U/mL penicillin (ThermoFisher), 100 mg/mL streptomycin (ThermoFisher), and cultured at 37°C in a 7.5% CO_2_ incubator. The culture media was replaced and the cells were checked by microscopy every other day. Colonies were trypsinized by 0.25% trypsin-EDTA solution (Gibco) for 5 min at 37°C and mechanically dissociated for passaging every 7–10 days. To harvest single-cell suspensions of CoSCs derived from passage 3–8 (P3–P8) cultures for sequencing, colonies were digested by TrypLE Express solution (Gibco) for 15 min at 37°C and passed through 30-µm filters (Miltenyi Biotec).

### Immunofluorescence staining

After differentiation of the 2D system of colon stem cells, the cells were fixed with a 4% formaldehyde solution for more than 30 min. The fixed samples were washed three times with PBS for 10–15 min each time. Prepare the closure buffer: Add 1 g of bovine serum albumin to each 20 mL of PBS buffer, then add 60 μL of TritonX-100 and mix well by vortex shaking. Add closure buffer to the sample and shake for 3 h at room temperature or overnight at 4°C. Prepare antibody dilutions: add 0.2 g of bovine serum albumin to each 20 mL of PBS buffer, then add 60 μL of TritonX-100. Aspirate and discard the blocking buffer, dilute the primary antibody proportionally with antibody dilutions, add to the wells, and incubate at 4°C overnight. After binding, the primary antibody was washed three times with PBS, and the fluorescent secondary antibody diluted with 5% BSA was added and bound at room temperature for 1.5 h. The nuclei were then stained with 4ʹ,6-diamidino-2-phenylindole (DAPI), washed three times with PBS, and photographed under an inverted fluorescence microscope.

### Immunoblot analysis

For evaluating protein expression in multiple CoSCs, cells were collected and lysed by sonication in RIPA lysis buffer containing 1 mM EDTA, 2 mM Na_3_VO_4_, 1 mM PMSF, and protease inhibitor cocktail (GE), and then subjected to 12% SDS–PAGE. The proteins were transferred to a PVDF membrane (Roche, USA). The membranes were blocked with 5% BSA in TBST buffer for 1 h at room temperature and then probed with indicated antibodies at 4°C overnight with gentle rotation. Using goat anti-mouse or anti-rabbit IgG HRP conjugate antibodies (Thermo, USA) as secondary antibodies, the proteins were visualized using enhanced chemiluminescence (Millipore, USA). Protein band densitometry was performed using ImageJ software (NIH, Bethesda, USA). At least three independent experiments have been carried out and representative results are shown.

### Antibodies

Antibodies used in this study were the following: anti-SOX9 (Cell Signaling Technology, 82630，1:200), anti-Ki67 (BD Biosciences, 550609, 1:200), anti-CHGA(Abcam,ab15160,1:200), anti-MUC2 (Santa Cruz Biotechnology,sc-515032,1:200), anti-rabbit IgG(H+L) cross-adsorbed Alexa Fluor 488(Thermo Fisher Scientific,A-21206, 1:500), anti-rabbit IgG(H+L) cross-adsorbed Alexa Fluor 594 (Thermo Fisher Scientific, A-21207, 1:500), anti-mouse IgG(H+L) cross-adsorbed Alexa Fluor 594 (Thermo Fisher Scientific, A-21203,1:500), anti-METTL3 antibody (Abcam, ab195352, 1:1000), anti-METTL14 antibody (Abcam, ab220030, 1:1000), anti-FTO antibody (Abcam, ab124892, 1:1000), and anti-YTHDC1 antibody (Abcam, ab122340, 1:1000).

### Sequencing library

#### RNA-seq and meRIP-seq

Total RNA was extracted from CoSCs using TRIzol reagent (Invitrogen) and the polyadenylated mRNA was purified with the Dynabeads mRNA purification kit (ThermoFisher). mRNAs (50–100 ng) were fragmented into ~150 nt by RNA fragmentation reagents (Invitrogen). Purified mRNA (1/20) was used as an input sample to construct the RNA library using SMARTER stranded total RNA-seq Kit V2 (Clontech) according to the manufacturer’s instructions. The remaining fragmented mRNA was immunoprecipitated with m^6^A antibody in the EpiMark *N*^6^-methyladenosine enrichment kit (NEB) to enrich the m^6^A-marked RNA. RNA was enriched through RNA Clean & Concentration-5 (Zymo Research) and the library was constructed with SMARTer Stranded Total RNA-seq Kit v2 (Clontech) for meRIP-seq. All libraries were purified using Agencourt AMPure® XP beads (Beckman Coulter, A63881) to obtain purified libraries with a primary peak of ~300bp. The RNA-seq and meRIP-seq libraries were sequenced using the Illumina NovaSeq PE150 platform.

#### Omni-ATAC-seq

Omni-ATAC-seq was performed according to an existing protocol [49] with some modifications. CoSCs (1 × 10^4^) were collected and permeabilized by adding 50 μL cold ATAC-RSB buffer (10 mM Tris–HCl pH 7.5, 10 mM NaCl, 3 mM MgCl_2_) containing 0.1% NP40, 0.1% Tween-20, 0.01% digitonin, and incubated on ice for 3 min. The cells were then washed with 1 mL cold ATAC-RSB containing 0.1% Tween-20, and the nuclei were pelleted by centrifugation. Nuclei were resuspended in 50 μL transposition mixture [25 μL 2× TD buffer (20 mM Tris–HCl pH 7.5, 10 mM MgCl_2_, 20% dimethyl formamide), 5 μL transposase, 16.5 μL PBS, 0.5 μL 1% digitonin, 0.5 μL 10% Tween-20, 2.5 μL H_2_O and incubate at 37°C for 30 min in a thermomixer with 1000 rpm mixing. The transposed DNA was cleaned with DNA Clean and Concentrator-5 Kit (Zymo Research) following the manufacturer’s instructions. The DNA library was prepared using TruePrep® DNA Library Prep Kit V2 for Illumina (Vazyme) doing five cycles of pre-amplification and one to four cycles of PCR amplification with NEBNext® Ultra™ II Q5® Master Mix (NEB). The samples were purified with two-sided size selection by Agencourt AMPure® XP Beads (Beckman Coulter, 0.5X and 0.5X reaction volume) and the DNA was dissolved in ~10 μL of nuclease-free water. The Omni-ATAC-seq libraries were subsequently sequenced using the Illumina NovaSeq PE150 platform.

#### Compliance with ethical guidelines

The colonic tissues were obtained from non-IBD individuals and UC patients at the General Hospital of the People’s Liberation Army, Tsinghua Changgung Hospital, and Peking Union Medical College Hospital. These acquisitions were approved by the Ethics Committees of Tsinghua University (20170019 and 20190303).

### Bioinformatic analysis

#### meRIP-seq and RNA-seq data analysis

FastQC v0.11.8 (https://www.bioinformatics.babraham.ac.uk/projects/fastqc/) was used for the quality control of raw sequencing data. Adaptor and low-quality bases were trimmed with Trim Galore version 0.6.0 and parameters “-q 20 --three_prime_clip_R1 3 --clip_R2 3 --phred33 –illumina --paired --stringency 3 --length 36”. The Fastq files were mapped into human Ribosomal RNA by Bowtie2 version 2.3.5 [50], the reads that did not align were retained and aligned on the hg38 human reference genome by STAR version 2.7 and default settings [51]. Reads with mapping quality above 20 were obtained with samtools version 1.7 [52, 53] and used for downstream analysis.

The m^6^A input data was considered as general RNAseq for analysis. The featureCounts function of Rsubread v2.0.1 was used to calculate the gene expression of the transcriptome [54], and genes with an average count ≤1 were filtered out. TPM normalization was based on sequencing depth and on the longest transcript length. Differential expression analysis was performed with DESeq2 v1.26.0 [55], and genes with adjusted *P-*value ≤ 0.05 and | log_2_(fold change) | ≥ 0.5 were considered as differentially expressed genes. The principal component analysis (PCA) was performed with the R function “prcomp”. PCA scatter plot was generated with the ggplot2 package. ClusterProfiler v3.14.3 [56, 57] was employed for functional enrichment analysis and GSEA analysis.

m^6^A peaks were identified with the exomePeak package [58, 59] and default parameters. The transcriptomic distribution of genomic features was plotted by the Guitar package [60]. The m^6^A ratio of m^6^A peak was defined as the CPM value of m^6^A peak divided by the gene expression value TPM of the corresponding gene: (CPM+1)/(TPM+1), the m^6^A bigwig file for visualization also used this normalization method. m^6^A peaks with a difference of m^6^A ratio above 0.3 were defined as “m^6^A gain peaks”. Peaks with a difference of m^6^A ratio <−0.3 were defined as “m^6^A loss peaks”.

#### ATAC seq data analysis

Sequencing quality control for ATAC-seq data was performed as described in the previous section. Fastq files were mapped on the hg38 human reference genome with Bowtie2 version 2.3.5. ATAC peak calling was performed using MACS2 v2.1.3 and parameters “--nomodel --shift -100 --extsize 200” [61]. The peaks from each sample were merged with bedtools v2.26.0 [62] and command “bedtools merge -d 20”. ATAC peaks were annotated with the perl script annotatePeaks.pl from the HOMER software [63]. The transcription start site (TSS) 1,000 bp upstream and 1,000 bp downstream was defined as “promoter-TSS” region. The featureCounts function of Rsubread v2.0.1 [54] was utilized to count reads on merged peaks. The peak counts were normalized to counts per million (CPM) using the edgeR packages [64–66] in R and the normalized factors were calculated with HouseKeeping peaks. The differential peak analysis was performed using exact tests for differences between two groups of Negative-Binomial Counts in edgeR. The HouseKeeping peaks were defined as the peaks located at “promoter-TSS” regions of colonic stem cells. HouseKeeping genes were obtained as follows: (i) Human HouseKeeping genes list were downloaded from Housekeeping and Reference Transcript Atlas (HRT Atlas v1.0, www.housekeeping.unicamp.br); (ii) In Human HouseKeeping genes list, genes whose expression values met the following criteria were identified as housekeeping genes for colonic stem cells [67]:

(i) The gene must be expressed at the non-zero level in all samples;
(ii) The standard deviation of the log_2_ TPM of the gene should be lower than 1 in all samples;
(iii) The maximum fold change (MFC), represented by the ratio between maximum and average log_2_ TPM of the transcript ((maximum log_2_ TPM)/(average log_2_ TPM)), should be lower than 2.

The bigwig format files of the ATAC signal were transformed from bam files with deepTools [68] based on BPM normalization in 1 bp bin size, multiplied by HouseKeeping peaks scaling factors, and then visualized with the IGV software [69]. The HouseKeeping peaks scaling factors were calculated as follows:


$$\begin{array}{l} \frac{10^{6}}{norm.factor*library.size} \end{array}$$

The normalization factor (*norm.factor*) and library size (*library.size*) were calculated with the calcNormFactors function from the edgeR package. The enhancer annotations were downloaded from the EnhancerAltas 2.0 [70].

#### Gene regulation cases analysis

To understand how the epigenome and epitranscriptome regulate RNA expression in CoSCs, we integrated the gene’s chromatin accessibility and m^6^A modification state information with mRNA expression data. Firstly, we defined four gene regulation cases: ① if the gene has no detectable chromatin accessibility region in the promoter-TSS region (TSS site ±1kb) and no m^6^A modification, we defined it as not regulated by ATAC and m^6^A modification “−−”; ② if the gene only has a chromatin accessibility peak in the promoter region but no m^6^A modification, we defined it as only regulated by chromatin accessibility, “+–”; ③ if the gene only presents m^6^A modification, we define it as only regulated by m^6^A modification, “−+”; ④ if a gene both has accessible chromatin in the promoter region and m^6^A modification, it’s defined as regulated by both chromatin accessibility and m^6^A modification, “++”.

To understand how changes in chromatin accessibility and m^6^A modification affect gene expression in the “++” group, we performed K-means analysis for chromatin accessibility log2foldchange and the difference in m^6^A ratio with the Cluster 3.0 software [71] and parameters “−g 7 –k 6 –r 1000”.

## Supplementary Material

lnad034_suppl_Supplementary_Figures

lnad034_suppl_Supplementary_Table_S1

lnad034_suppl_Supplementary_Data

## Data Availability

The sequencing data have been deposited in Genome Sequence Archive for human (GSA-human) repository with the accession number HRA003840.
