# Supplementary figures and images for: Colonic stem cell from severe ulcerative colitis maintains environment-independent immune activation by altering chromatin accessibility and global m6A loss
Source: Life Med. 2023 Sep 13;2(4):lnad034. doi: 10.1093/lifemedi/lnad034 (PMC11749566; doi:10.1093/lifemedi/lnad034)

Figure S1

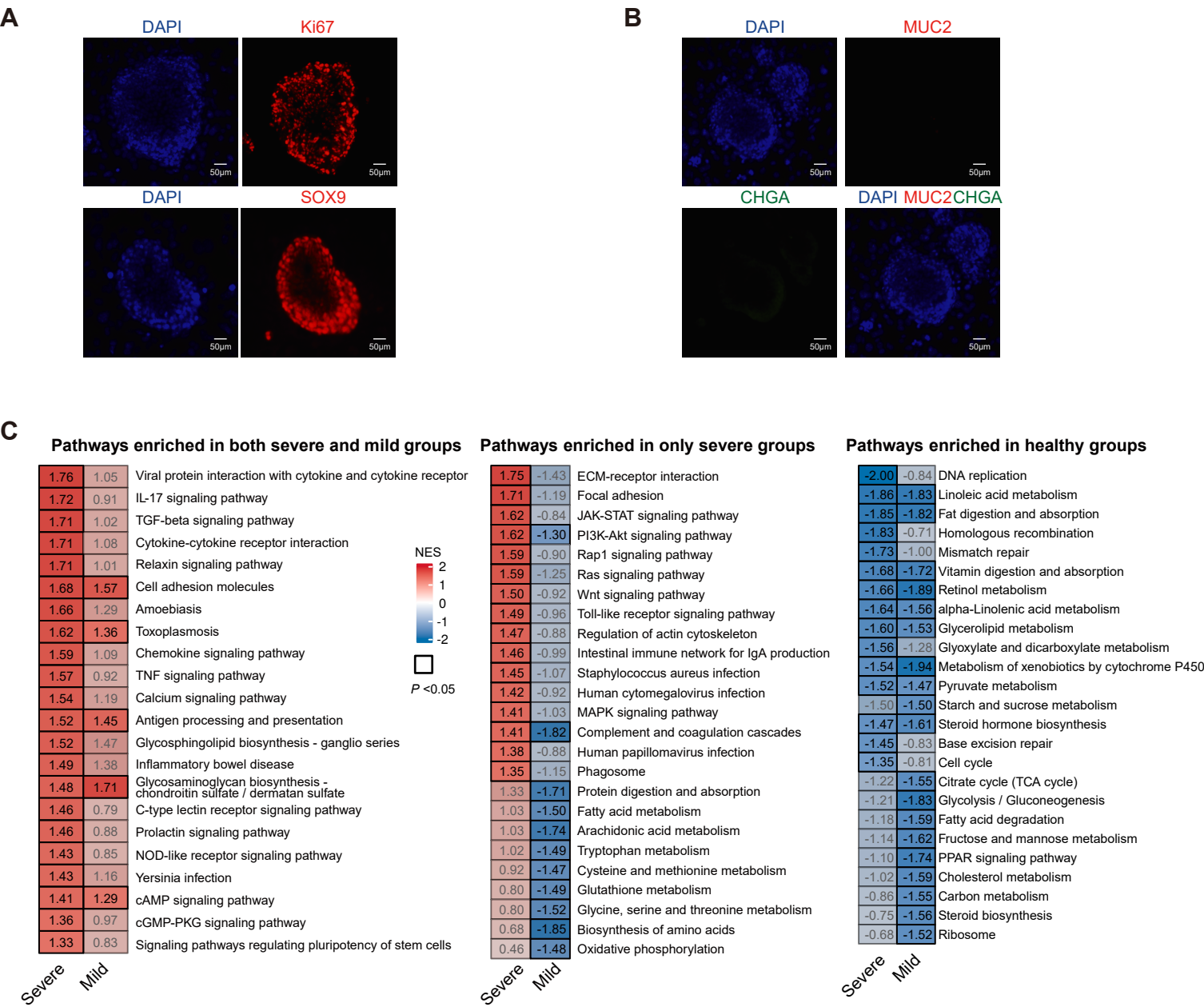

Figure S2

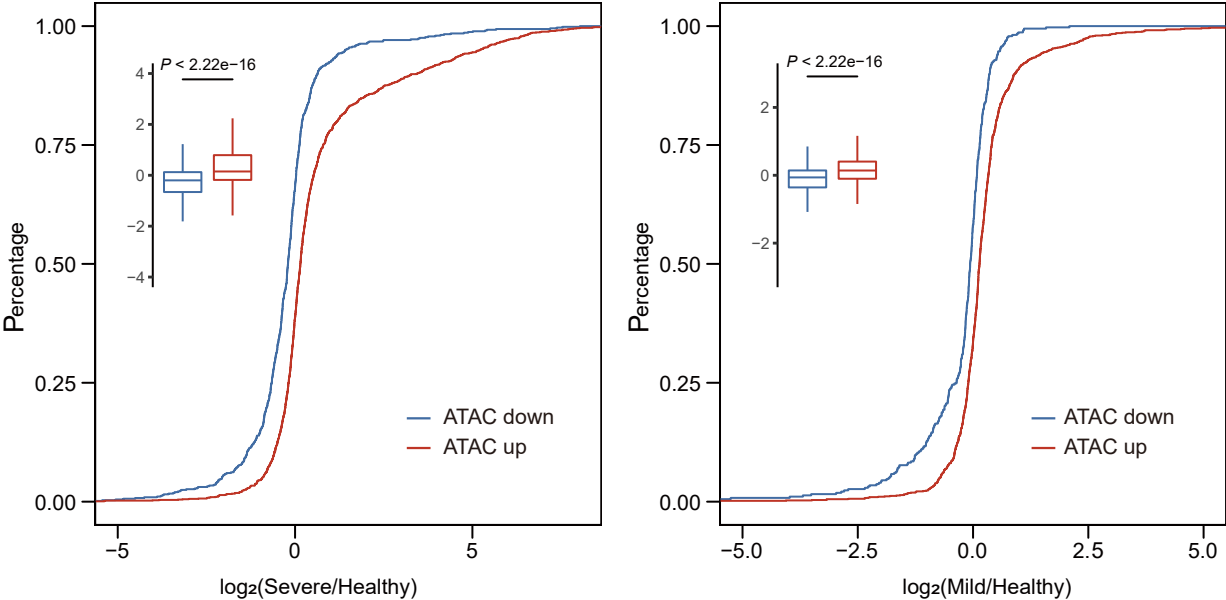

Figure S3

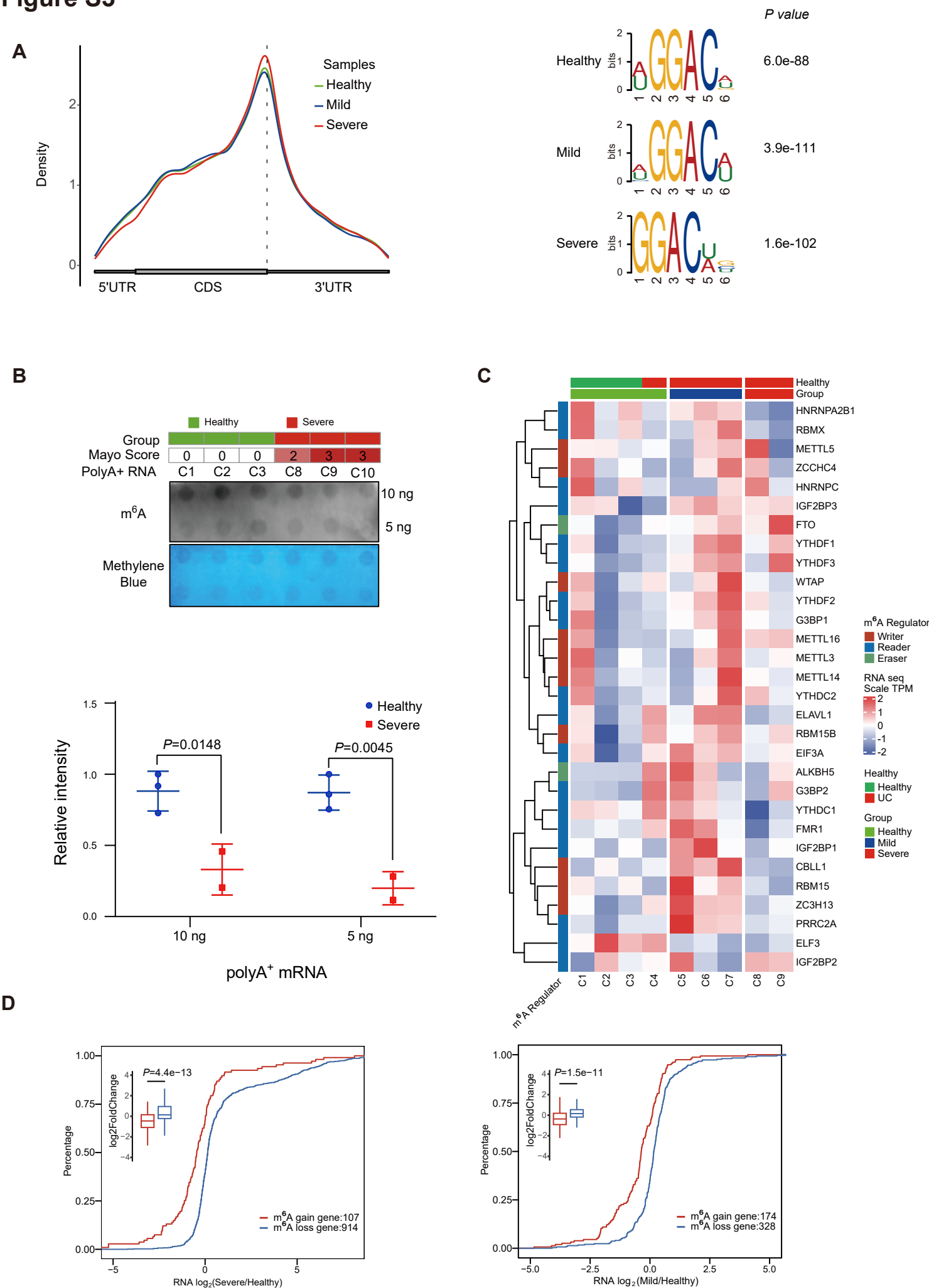

Figure S4

A

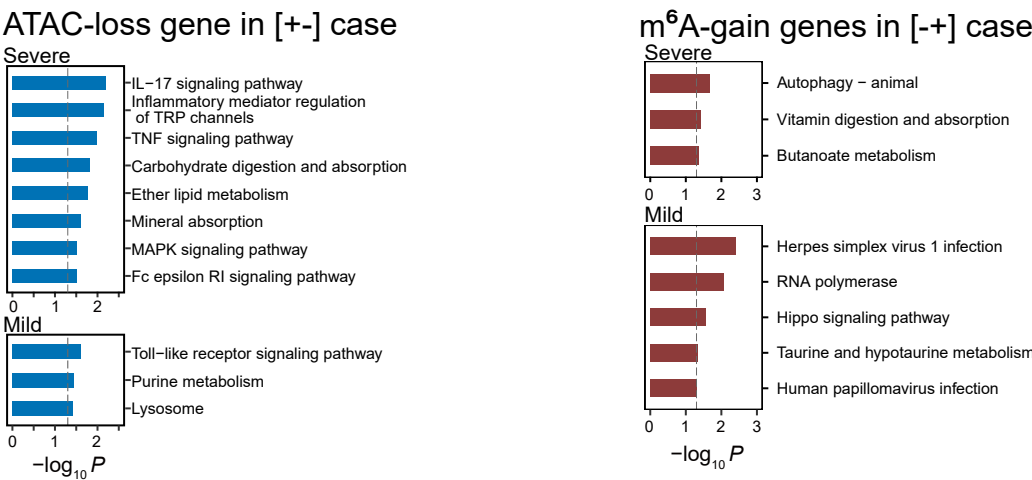

B

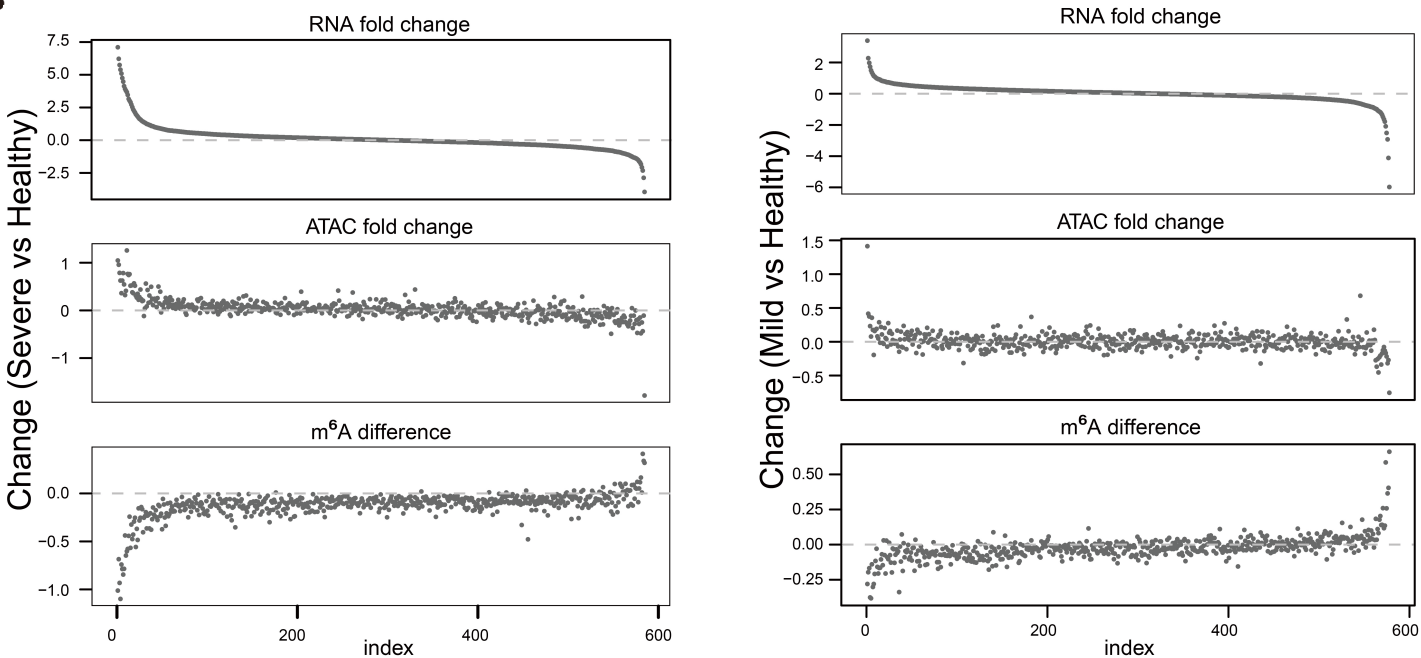

C

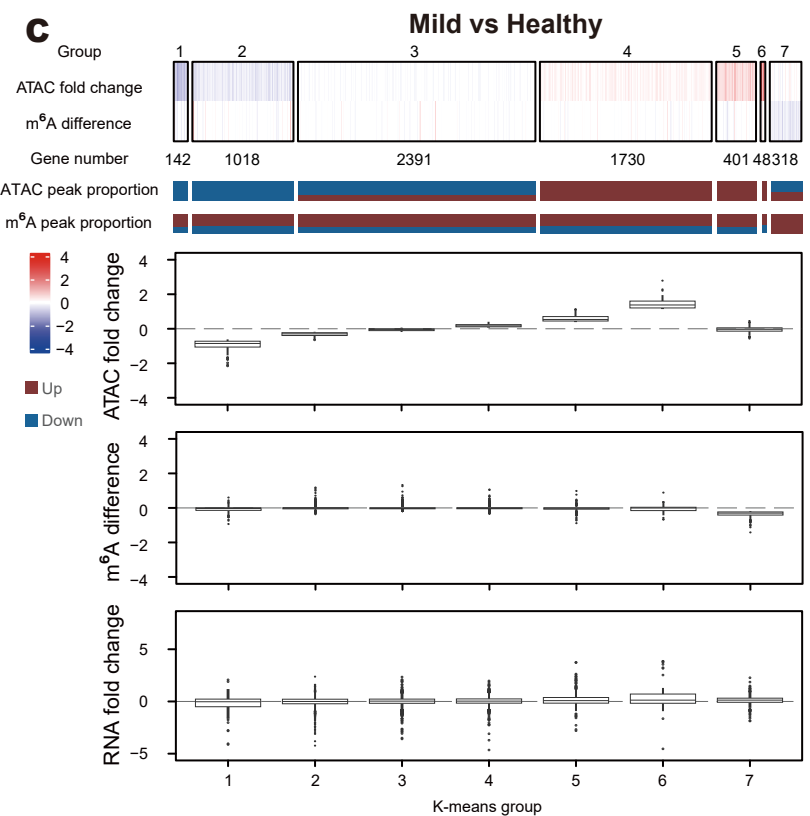

D

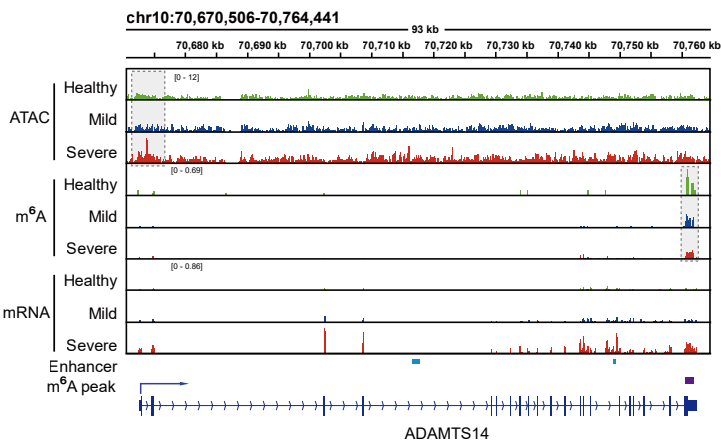

Supplement: lnad034_suppl_Supplementary_Figures [file lnad034_suppl_Supplementary_Figures.pdf]
