# Supplementary material for: Colonic stem cell from severe ulcerative colitis maintains environment-independent immune activation by altering chromatin accessibility and global m6A loss
Source: Life Med. 2023 Sep 13;2(4):lnad034. doi: 10.1093/lifemedi/lnad034 (PMC11749566; doi:10.1093/lifemedi/lnad034)
Supplement: lnad034_suppl_Supplementary_Data [file lnad034_suppl_Supplementary_Data.docx]

**Supplementary Figure Legend**

**Figure S1. Enriched pathways in the differentially expressed genes in severe and mild UC**

1. Representative staining of CoSCs colonies stained with indicated antibodies for proliferative marker Ki-67 or colon epithelial stem cells marker SOX9. Scale bar, 50 μm.
2. Representative staining of CoSCs colonies stained with indicated antibodies for intestinal goblet cell marker MUC2 and endocrine cell marker CHGA. Scale bar, 50 μm.
3. GSEA analysis visualizing differences in enriched pathways between mild and severe UC. Colors indicate the NES scores. A black box around the value indicates *P*-value<0.05.

**Figure S2. Chromosome accessibility and m^6^A modifications have major but different variations in severe and mild UC patients.**

Cumulative curves demonstrating the distribution of expression changes of genes with up- and down-regulated ATAC signaling, where expression change values are defined as log-transformed fold change. The boxplot shows the difference between the two types of gene expression changes. The left panel shows severe UC compared to healthy individuals, while the right panel shows mild UC compared to healthy individuals.

**Figure S3. RNA m^6^A modifications present a global loss in UC patients.**

1. Meta gene plot showing the characteristic distribution of m^6^A on the transcriptome. The small SeqLogo plots show the m^6^A feature motif enriched on the m^6^A peak.
2. The levels of mRNA m^6^A in CoSCs of healthy individuals or patients with severe UC with mRNA dot blot. Methylene blue staining was used as a loading control. Relative m^6^A intensity were evaluated in right panel. Data were analyzed using two-tailed unpaired t test.
3. Heatmap showing the expression profile of known RNA m^6^A reader, writer and eraser proteins.
4. Cumulative curves demonstrating the distribution of expression changes of genes with up- and down-regulated normalized m^6^A signaling, where expression change values are defined as log-transformed fold change. The boxplot shows the difference between the two types of gene expression changes. The left panel shows severe UC compared to healthy individuals, while the right panel shows mild UC compared to healthy individuals.

**Figure S4. RNA m^6^A and chromatin accessibility determine the abnormality of the downstream transcriptome in UC patients.**

1. Left, bar plots showing pathways enriched by ATAC-loss genes in severe and mild UC patients compared to the healthy individuals on genes with open promoters and no m^6^A signal. Right, bar plots showing pathways enriched by the m^6^A-gain genes in severe and mild UC patients compared to the healthy individuals on genes with m^6^A signal at gene body regions and no ATAC signal at promoters. Values are negative log10 transformed *P*-values.
2. Scatter plots representing the fold change of RNA expression, ATAC signal and m^6^A signal difference of genes co-regulated by m^6^A and chromatin accessibility at promoter regions. The genes are ranked by fold change of expression level from the highest to the lowest, and 10 genes were grouped into a bin to calculate the mean values of RNA expression, ATAC signal and m^6^A signal. The x axis is the rank index.
3. Genes co-regulated by m^6^A and chromatin accessibility at promoter regions classified into seven groups by k-means clustering shown in the upper heatmap. The middle bar graph shows the percentage of up- or down-regulation of m^6^A or ATAC signals on genes in each clustering group. The boxplot below presents the difference values of m^6^A signal and fold changes of ATAC signal and RNA expression in each group of genes in mild UC patients compared to the healthy ones.
4. IGV tracks displaying RNA and m^6^A abundances of ADAMTS14 transcripts in CoSCs.

**Table S1.** Detailed information of clinical parameters and multi-omics application used in the present study.
